# Sedentary time and its association with risk of cardiovascular diseases in adults: an updated systematic review and meta-analysis of observational studies

**DOI:** 10.1186/s12889-022-12728-6

**Published:** 2022-02-12

**Authors:** Wu Jingjie, Lili Yang, Ye Jing, Lulu Ran, Xu Yiqing, Na Zhou

**Affiliations:** 1 School of Medicine, Zhejiang University, 268th of Kaixuan Road, Shangcheng District, Hangzhou, Zhejiang Province China; 2 Nursing Department, The Fourth Affiliated Hospital, Zhejiang University, School of Medicine, N1 of Shangcheng Avenue, Yiwu, Zhejiang Province China; 3 School of Allied health professions, Loma Linda University, 24951 N Circle Drive, Loma Linda, CA 92350 USA; 4 Sir Run Run Shaw Hospital, School of Medicine, Zhejiang University, 3th of East Qingchun Road, Shangcheng District, Hangzhou, Zhejiang Province China

**Keywords:** Sedentary time, Screen time, Cardiovascular diseases, Morbidity, Mortality, Systematic review, Meta-analysis

## Abstract

**Background:**

Epidemiological studies assessing the association between sedentary time and cardiovascular diseases (CVD) risks have been published at a rapid pace in recent years, which makes the periodic review of knowledge essential. Furthermore, much of the early and ongoing work used screen time as a marker of total sedentary time, which may weaken the association between sedentary time and CVD risks.

**Objective:**

To update evidence on CVD risks associated with different types of sedentary time, especially total sedentary time and screen time, and to explore as a marker of total sedentary time, whether screen time had similar CVD risks with total sedentary time.

**Methods:**

PRISMA guideline was followed for the performing and reporting of this systematic review and meta-analysis. Three independent researchers searched eight electronic databases and two clinical trial registries for all studies published between January 2015 and December 2021 that assessed the association between sedentary time and CVD risks in adults. A standardized form was used for data extraction and collection. Wilmot and colleagues’ modified tool was used for quality assessment. The categorical association was assessed by comparing the pooled effect sizes for CVD risks associated with the highest and the lowest sedentary time categories across included studies. Stata 16.0 and Review Manager 5.3 were used for all statistical analyses, *P* ≤ 0.05 was considered as statistically significant.

**Results:**

Seventeen prospective cohort studies and two cross-sectional studies with 145,1730 participants and over 48,668 CVD cases and deaths were included. Two included studies measured sedentary time with the accelerometer, 16 studies with self-reported questions, and one study with both the accelerometer and self-reported questions. CVD outcomes were self-reported in two included studies and objectively adjudicated through medical records or death certifications in 17 studies. Compared with the lowest total sedentary time category (median duration, 2.75 h/d), participants in the highest category (median duration, 10.5 h/d) had an increased risk of CVD morbidity (pooled RR, 1.24; 95% CI, 1.21–1.27). Compared with the lowest total sedentary time category (median duration, 2.98 h/d), participants in the highest category (median duration, 10.2 h/d) had an increased risk of CVD mortality (pooled HR, 1.29; 95% CI, 1.13–1.47). The association between screen time and CVD risks was similar to total sedentary time with the cut-off point of 5–6 h/d. The associations between occupational sitting time, leisure sedentary time, and CVD risks stayed inconclusive.

**Conclusion:**

Total sedentary time and screen time are both associated with cardiovascular health. As a marker of total sedentary time, screen time over 5–6 h/d had similar CVD risks with total sedentary time over 10–11 h/d.

**Supplementary Information:**

The online version contains supplementary material available at 10.1186/s12889-022-12728-6.

## Introduction

Cardiovascular diseases (CVD) are the leading cause of death globally, taking an estimated 17.9 million lives each year [1, 2]. Physical inactivity is a well-known and modifiable risk factor for CVD. Insufficient moderate-to-vigorous physical activity and prolonged sedentary behavior are two independent facets of physical inactivity. Current guidelines regard exercise as a cornerstone in maintaining and improving cardiovascular health and recommend at least 150 min of moderate physical activity or 75 min of vigorous physical activity per week to reduce the CVD risks [3, 4]. Despite these recommendations, more than half of adults do not meet the minimum volume of physical activity based on the data from the Chinese National Center for Cardiovascular Diseases and the American College of Cardiology [2, 3]. For older people and people with physical disabilities, meeting these recommendations can be particularly burdensome or even impossible. Thus, as another facet of physical inactivity, more attention should be paid to sedentary behavior and its preventive interventions, especially for people who spend most of their waking time sedentarily and cannot meet the recommended level of physical activity.

Sedentary behavior is defined as any waking behavior characterized by an energy expenditure≤1.5 metabolic equivalents (METs) while in a sitting, reclining or, lying posture [5], and sedentary time is the time spent in sedentary behavior. A dose-response meta-analysis published in 2016, including 72,0425 participants and 25,769 CVD cases and deaths, found a nonlinear association between total sedentary time and risk of CVD in continuous analysis, with a statistically significant increased risk observed only at a total sedentary time of more than 10.04 h/d [6]. In recent years, epidemiological studies assessing the association between sedentary time and CVD risks have been published at a rapid pace, especially prospective cohort studies with large sample sizes and long follow-up durations, which makes the periodic review of knowledge essential. Furthermore, much of the early and ongoing work in this area used screen time as a marker of total sedentary time [7]. Since sitting in front of electronic screens is one of the sedentary behavior types, representing screen time as a marker of total sedentary time may weaken the association between total sedentary time and CVD risks. However, to the best of our knowledge, there is no previous study exploring whether screen time is a real marker of total sedentary time, which means having similar CVD risks. To fill these gaps, the primary objective of this study was to update CVD risks associated with different types of sedentary time, especially total sedentary time and screen time. The second objective was to explore as a marker of total sedentary time, whether screen time had similar CVD risks to total sedentary time.

## Methods

### Literature Search Strategy

The Preferred Reporting Items for Systematic reviews and Meta-Analyses (PRISMA) guidelines was followed for the performing and reporting of this present systematic review and meta-analysis (Supplementary File 1) [8, 10]. We searched for all observational studies including prospective cohort studies, retrospective case-control studies, and cross-sectional studies, that assessed the association between sedentary time and CVD risks among adults (over 18 years old at baseline). Systematic searches through eight electronic databases including PubMed, Embase, Cochrane Library, Web of Science, China National Knowledge Infrastructure, Wanfang, Weipu, and SinoMed, and two clinical trial registries including Clinical trials and China Clinical Trial Registry, were performed with the limitation of publication date between January 2015 and December 2021. We used various combinations of the following keywords and MeSH terms: sedentary behavior, sedentary lifestyle, screen time, television, computers, video games, occupational sitting, cardiovascular diseases, coronary disease, stroke, mortality, morbidity, risk factors, et al. Detailed search terms are shown in Supplementary File 2 (Table S1). Reference lists of the included studies and relevant reviews were also manually searched.

### Study Selection

The primary exposure indicator of interest in this study was total sedentary time, defined as total waking hours per day that have low energy expenditure and are often performed in a sitting or reclining posture. The secondary exposure indicator was screen time, defined as total waking hours per day that are in front of an electronic screen, such as watching TV, using personal computers in leisure time, or playing video games, considering that as a marker of total sedentary time, it has been demonstrated to be associated with CVD risks in population cohort studies [7, 9, 11]. Additional exposure indicators such as occupational sitting time and leisure sedentary time were considered tertiary. The primary outcome in this study was CVD risks (morbidity or mortality), including ischemic heart diseases (IHD), coronary heart diseases (CHD), stroke, heart failure and CVD-related death. Because several original studies’ outcome was CVD morbidity and mortality, it was considered as the second outcome.

Observational studies that assessed CVD risks associated with all types of sedentary time were included in the initial study selection process, but only studies that reported total sedentary time or screen time as the main exposure indicator were included in the meta-analysis. Three independent reviewers (JJW, LLR & JY) performed the initial screening of all titles and abstracts and then evaluated all potentially relevant articles based on the full-text reviews. Studies were excluded if they failed to meet all the criteria mentioned above. Any discrepancies regarding study inclusion were adjudicated by discussion, and if needed, in consultation with the senior author (LLY). If multiple studies used the data of the same sample source, studies with longer follow-up durations, larger sample sizes, and/or more detailed reports of sedentary behavior and physical activity were included.

### Quality Assessment

The study quality was assessed using the methods created by Wilmot and colleagues [10], which had similar elements with Newcastle-Ottawa Scale but were modified to assess studies that explore sedentary time with associated risks for health problems. The total score of this quality assessment tool was 6 (1 for a prospective study design; if sedentary time was self-reported, 1 for reported reliability, 1 for reported validity, 2 if an objective measurement of sedentary time was used; 1 if two or more covariates were adjusted for; 1 if physical activity was adjusted for; 1 for an objective measurement of CVD outcomes, such as medical records and death certifications). A score of 5 or 6 was considered as high quality, 3 or 4 moderate quality, and 0 or 2 poor quality. Studies attaining poor quality were excluded from this study.

### Data Collection

Three independent reviewers (JJW, LLR & JY) independently performed the data collection using a standardized form including publication information (first author’s family name and publication year), country, sample source, study design, follow-up duration, sample characteristics (size, age, and proportion of male), types, definitions and measurements of sedentary time, physical activity and CVD outcomes, the number of cases, odds ratio (OR), hazard risk (HR), relative risk (RR) and confidence interval (CI) extracted from the most adjusted models and covariates that entered the most adjusted models. Any discrepancies regarding extracted data were resolved by discussion, and if needed, in consultation with the senior author (LLY).

### Statistical analysis

#### Exposure Assessment

For studies that assessed categorical associations between sedentary time and risk of CVD, all of them reported quantitative estimates as average duration or time range. The average duration for each category was used to define the median sedentary time for that category. If a time range was reported for a sedentary time category without the average duration, we estimated the approximate median sedentary time using the midpoints of the lower and upper boundaries. For studies with an open-ended highest sedentary time category, we assumed that this category had the same amplitude as the closet category. For studies with an open-ended lowest sedentary time category, we assumed that the lower boundaries of this category were 0 h/d. As for studies that assessed the continuous association between sedentary time and risk of CVD, and reported effect size as each additional hour of sedentary time, we assumed that the reference category was 1 h/d, and the other category was 2 h/d. The reported time and estimated median of each sedentary time category are detailed in Supplementary File 2 (Table S2).

#### Outcome Assessment

We extracted HRs and 95% CIs from the Cox proportional hazard regression models and extracted RRs and 95% CIs from the logistic regression models. All these models had the most complete adjustment for other CVD risk factors. Where RR and 95% CI were not given, a formula from Zhang and Yu was used to correct the most adjusted OR ($RR=\frac{OR}{\left(1-P0\right)+\left(P0\times OR\right)}$_,_ P0 indicates the incidence of the outcome in the non-exposed group) [12]. We considered HRs to be closely equal to RRs and used RRs to assess the association between sedentary time and CVD risks.

#### Categorical Analysis

Because most of the included studies had two or more sedentary time categories, we used a previously described approach to pool data across studies and generated two pooled categories of sedentary time (the highest and the lowest) as described in Table 2 [6]. The pooled RRs and 95% CIs for CVD risks associated with different types and categories of sedentary time were calculated by comparing the highest with the lowest categories. Heterogeneity of RRs and 95% CIs across studies was tested by using the *I*^2^ statistics at the *P*<0.10 level of significance, when *I*^2^ ≤ 50% and *P*>0.10, Inverse-Variance fixed-effect model was used to pool the RRs and 95% CIs, whereas DerSimonian and Laird random-effect model was used. Sensitivity analysis was further performed to examine the influence of various exclusion criteria on the overall risk estimate. Publication bias was assessed by Egger’s and Begg’s tests. Sensitivity analysis and part of publication bias were performed using Stata 16.0. Other analyses were performed using Review Manager version 5.3. Statistical analyses were declared significant for a two-sided *P* ≤ 0.05.

## Results

### Search Results and Study Characteristics

The study selection process and results are shown in Supplementary File 2 (Fig. S1). Briefly, 6813 studies were identified through searching the electronic databases and clinical trial registries, and two studies were added through hand-searching from reference lists of the included studies. A study reporting an inappropriate OR value of 176.62 (95% CI: 43.33–719.90) was identified as an outlier and excluded after discussion. Finally, inclusion criteria were met in 19 studies [7, 9, 11, 13–28], including 17 prospective studies and two cross-sectional studies with 145,1730 unique participants and over 48,668 unique CVD cases and deaths. Baseline characteristics of the included studies are shown in Table 1. Of the 19 included studies, six were from America, five were from Europe, and four each from Asia and Australia. One study included only women and 18 studies included men and women. Nine studies’ exposure indicator was total sedentary time (or total sitting time), nine studies were screen time including time spent in television watching, computer using in leisure time and video gaming, two studies were occupational sitting time, and one study was leisure sedentary time. Seven studies’ outcome was CVD morbidity, nine was CVD mortality, and four was CVD morbidity and mortality. Total sedentary time was assessed with self-reported questions in five studies and assessed with the accelerometer in three studies. Screen time, occupational sitting time, and leisure sedentary time were all assessed with self-reported questions. CVD outcomes were adjudicated by self-report in two studies, and by medical records or death certifications came from the administrative database and/or national mortality index in 17 studies (Supplementary File 2, Table S4). Most studies adjusted for covariates such as age (*n* = 17), sex (*n* = 17), physical activity (*n* = 17) and smoking (*n* = 17) in the most adjusted model (Supplementary File 2, Table S3a, S3b, S3c). Assessment of study quality yielded an average score of 4.79, and a median score of 5 (Supplementary File 2, Table S5). The main reasons for not scoring were as follows: (i) the self-reported questions used to measure sedentary time without a reliability or validity test; (ii) the study design was a cross-sectional study; (iii) physical activity was not included in the most adjusted models as a covariate; and (iv) CVD outcomes were self-reported.

**Table 1:** Baseline Characteristics of the Studies Included in the Systematic Review and Meta-analysis

Publication	Country	Sample Source	Study Design	Follow-up Duration, years	Sample Size	Age, years	Male, %	Exposure indicator	CVD Outcomes	
									Assessed	Cases
Park, 2021 [14]	South Korean	KNHANES (2014–2018)^a^	Cross-sectional study	0	6785	≥65	44.57	Total sedentary time	CVD morbidity	914
Hamer, 2020 [11]	UK	UK Biobank (2006–2010)^a^	Prospective study	10.4	479,658	56.5 ± 8.0	45.7	Screen time	CVD mortality	1437
Liu, 2020 [13]	China	China-PAR (1998, 2000–2001, 2007–2008)^a^	Prospective study	5.8	93,110	52.8 ± 12.3	39.4	Total sedentary time	CVD morbidity or CVD mortality	3799
Tu, 2020 [18]	China	Self-sampling	Prospective study	5.9	3019	≥18	42.9	Total sedentary time	CVD morbidity and mortality	143
Bellettiere, 2019 [19]	US	OPACH (2012–2014)^a^	Prospective study	4.9	5638	79.0 ± 7.0	0	Total sedentary time	CVD morbidity and mortality	NA
Garcia, 2019 [21]	US	JHS (2000–2004)^a^	Prospective study	8.4	3592	≥21	36.7–40.8	Screen time & Occupational sitting time	CVD morbidity	168
Stamatakis, 2019 [15]	Australia	The 45 and Up study (2006–2009)^a^	Prospective study	7.4	148,836	≥45	44.5	Total sitting time	CVD mortality	1403
Patel, 2018 [26]	US	CPS-II Nutrition study (1992)^a^	Prospective study	20.3	127,554	50–74	44.38	Leisure sedentary time	CVD mortality	16,083
Morales, 2018 [9]	UK	UK Biobank (2007–2010)^a^	Prospective study	4.1	205,338	40–69	NA	Screen time	CVD morbidity	9660
Dohrn, 2017 [16]	Sweden	ABC study (2001–2002)^a^	Prospective study	14.2	851	66.7 ± 10.2	44	Total sedentary time	CVD mortality	24
Engelen, 2017 [27]	Australia	ANNPAS (2011–2012)^a^	Cross-sectional study	0	9435	≥18	NA	Total sitting time	CVD morbidity	NA
Cumming, 2016 [22]	Australia	AusDiab (1999–2000)^a^	Prospective study	12	9104	50.7 ± 13.4 to 67.4 ± 13.0	44.33	Screen time	CVD morbidity	126
Evenson, 2016 [17]	US	NHANES (2003–2006)^a^	Prospective study	6.7	3809	55.3	45.4	Total sedentary time, screen time	CVD mortality	107
Grace, 2016 [24]	Australia	AusDiab (1999–2000)^a^	Prospective study	13.6	8907	≥18	44.3	Screen time	CVD mortality	209
McDonnell, 2016 [23]	US	REGARDS (2003–2007)^a^	Prospective study	7.1	22,257	≥45	44.5	Screen time	CVD morbidity	727
Moller, 2016 [28]	Denmark	DWECS (1990)^a^	Prospective study	12.2	11,996	18–59	NA	Occupational sitting time	CVD morbidity and mortality	510
Borodulin, 2015 [20]	Finland	National FINRISK 2002 study (2002)^a^	Prospective study	8.6	4516	47.0 ± 13.0	44.5	Total sedentary time	CVD morbidity and mortality	183
Ikehara, 2015 [25]	Japan	JACC study (1988–1990)^a^	Prospective study	19.2	85,899	40–79	41.9	Screen time	CVD mortality	5835
Keadle, 2015 [7]	US	NIH-AARP Health Study (1995–1996)^a^	Prospective study	14.1	221,426	50–71	57	Screen time	CVD mortality	7340

^a^Baseline years of the data collection

*Abbreviations*: *CVD* Cardiovascular Diseases, *KNHANES* the Korean National Health And Nutrition Evaluation Study, *UK* the United Kingdom, *US* the United States, *China-PAR* the Prediction for Atherosclerotic Cardiovascular Disease Risk in China project, *OPACH* the Objective Physical Activity and Cardiovascular Health Survey, *NA* Non-available, *JHS* The Jackson Heart Survey, *CPS-II* Cancer Prevention Study II, *ABC study* the Sweden Attitude Behavior and Change study, *ANNPAS* Australia Nutrition and Physical Activity Survey, *AusDiab* Australian Diabetes, Obesity and Lifestyle Study, *NHANES* The National Health and Nutrition Examination Survey, *REGARDS* The REasons for Geographic and Racial Differences in Stroke, *DEWCS* Danish Work Environment Cohort Study, *JACC* The Japan Collaborative Cohort Study for Evaluation of Cancer Risk, *NIH-AARP* The National Institutes of Health-American Association of Retired Persons Diet and Health Study

### Association Between Total Sedentary Time and CVD risks

Because Engelen’s study (OR, 1.28; 95% CI, 1.02–1.60) lacked the data needed to correct the most adjusted OR, this cross-sectional study was not included in the meta-analysis. Table 2 showed that there were one prospective study, and one cross-sectional study entered the meta-analysis to assess the association between total sedentary time and CVD morbidity. Compared with the lowest total sedentary time category (median duration, 2.75 h/d), participants in the highest total sedentary time category (median duration, 10.5 h/d) had an increased risk of CVD morbidity (pooled RR, 1.24; 95% CI, 1.21–1.27). No significant heterogeneity was observed in the pooled analysis across studies (*I*^2^ = 0%, *P* = 0.42). Because only two studies were included in this subgroup of meta-analysis, publication bias tests and sensitivity analysis were not suitable.

**Table 2:** Pooled Association Between Highest versus Lowest Sedentary Time Duration and CVD Risks

Type of Sedentary Time	Median duration of sedentary time, h/d	CVD outcomes	Pooled HR/RR [95% CI], *P* value	Heterogeneity test (*I*^2^ and *P* value)	Included Studies
Total sedentary time	Highest: 10.5 Lowest: 2.75	CVD morbidity	1.24 [1.21, 1.27] *P*<0.05	*I*^2^ = 0%, *P* = 0.42	Liu, 2020 [13]; Park 2021 [14]
	Highest: 10.2 Lowest: 2.98	CVD mortality	1.29 [1.13, 1.47] *P*<0.05	*I*^2^ = 35%, *P* = 0.20	Liu, 2020 [13]; Stamatakis, 2019 [15]; Dohrn, 2017 [16]; Evenson, 2016 [17]
	Highest: 5 Lowest: 1	CVD morbidity and mortality	1.29 [0.93, 1.80] *P* = 0.13	*I*^2^ = 69%, *P* = 0.04	Tu, 2020 [18]; Bellettiere, 2019 [19]; Borodulin, 2015 [20]
Screen time	Highest: 3.75 Lowest: 1	CVD morbidity	1.03 [0.99, 1.07] *P* = 0.21	*I*^2^ = 95%, *P*<0.001	Garcia, 2019 [21]; Morales, 2018 [9]; Cumming, 2017 [22]; McDonnell, 2016 [23]
	Highest: 5 Lowest: 1	CVD morbidity^a^	1.22 [0.98, 1.52] *P* = 0.07	*I*^2^ = 0%, *P* = 0.35	Garcia, 2019 [21]; McDonnell, 2016 [23]
	Highest: 5 Lowest: 1	CVD morbidity^b^	1.04 [1.03, 1.05] *P*<0.05	*I*^2^ = 32%, *P* = 0.23	Garcia, 2019 [21]; Morales, 2018 [9]; McDonnell, 2016 [23]
	Highest: 5 Lowest: 1	CVD mortality	1.27 [1.05, 1.52] *P* = 0.01	*I*^2^ = 87%, *P*<0.001	Hamer, 2020 [11]; Evenson, 2016 [17]; Grace, 2016 [24]; Ikehara, 2015 [25]; Keadle, 2015 [7]
	Highest: 6 Lowest: 1	CVD mortality^b^	1.36 [1.03, 1.78] *P*<0.05	*I*^2^ = 80%, *P* = 0.002	Evenson, 2016 [17]; Grace, 2016 [24]; Ikehara, 2015 [25]; Keadle, 2015 [7]

^a^Results of sensitivity analysis

^b^In these meta-analysis, studies which did not include any physical activity intensity in the most adjusted models as a covariate were excluded

There were four prospective studies entered in the meta-analysis to assess the association between total sedentary time and CVD mortality. Participants in the highest total sedentary time category (median duration, 10.2 h/d) had an increased risk of CVD-related death (pooled HR, 1.29; 95% CI, 1.13–1.47) when compared with the lowest total sedentary time category (median duration, 2.98 h/d). No significant heterogeneity or significant publication bias was observed in the pooled analysis across studies (*I*^2^ = 35%, *P* = 0.20; *P* for Egger line regression test = 0.144; *P* for Begg rank correlation test = 0.308). For sensitivity analysis, the exclusion of any single study did not materially change the overall risk estimates.

There were also three prospective studies that assessed total sedentary time with CVD morbidity and mortality. Compared with the lowest total sedentary time category (median duration, 1 h/d), participants in the highest total sedentary time category (median duration, 5 h/d) had an increased risk of CVD morbidity and mortality (pooled HR, 1.29; 95% CI, 0.93–1.80). However, this association was not statistically significant (*P* = 0.13). Moderate heterogeneity was observed in the pooled analysis across studies (*I*^2^ = 69%, *P* = 0.04). No significant publication bias was observed (*P* for Egger line regression test = 0.182; *P* for Begg rank correlation test = 0.296). For sensitivity analysis, the exclusion of any single study did not materially change the overall risk estimates. The forest plots and results of sensitivity analysis are shown in Supplementary File 2 (Fig. S2-S6).

### Association Between Screen Time and CVD risks

Table 2 showed that there were four prospective studies entered the meta-analysis to assess the association between screen time and CVD morbidity. Compared with the lowest screen time category (median duration, 1 h/d), participants in the highest screen time category (median duration, 3.75 h/d) had an increased risk of CVD (pooled HR, 1.03; 95% CI, 0.99–1.07). However, this association had no significant difference (*P* = 0.21). High heterogeneity was observed in the pooled analysis across studies (*I*^2^ = 95%, *P*<0.001). No significant publication bias was observed (*P* for Egger line regression test = 0.345; *P* for Begg rank correlation test = 0.734). For sensitivity analysis, exclusion of Cumming 2017 and Morales 2018 did materially change the overall risk estimates (pooled HR, 1.22; 95% CI: 0.98–1.52; *I*^2^ = 0%, *P* = 0.35). This association also had no significant difference (*P* = 0.07).

As for the association between screen time and CVD mortality, there were four prospective studies and one cross-sectional study entered in the meta-analysis. Participants in the highest screen time category (median duration, 5 h/d) had an increased risk of CVD-related death (pooled RR, 1.27; 95% CI, 1.05–1.52) when compared with the lowest screen time (median duration, 1 h/d). High heterogeneity was observed in the pooled analysis across studies (*I*^2^ = 87%, *P*<0.001). No significant publication bias was observed (*P* for Egger line regression test = 0.267; *P* for Begg rank correlation test = 0.462). For sensitivity analysis, the exclusion of any single study did not materially change the overall risk estimates.

These associations were further strengthened when two studies did not include any physical activity intensity in the most adjusted models as a covariate were excluded (Table 2). The forest plots and results of sensitivity analysis are shown in Supplementary File 2 (Fig. S8-S13).

### Association Between Other Types of Sedentary Time and CVD risks

There were two prospective studies that assessed occupational sitting time with the associated risk of CVD. Garcia found that comparing participants who never or seldom sat during working time, participants often or always sat during the working time had an increased risk of CVD morbidity (HR, 1.06; 95%CI, 0.73–1.55). Adversely, Moller found that comparing participants who had more than 25 h/week being seated at work, participants who had less than 25 h/week being seated at work had a decreased risk of CVD morbidity and mortality (HR, 0.94; 95%CI, 0.71–1.27). Both associations had no significant difference. There was one study that assessed leisure sedentary time with the associated risk of CVD mortality. Patel found that comparing participants who had less than three hours per day being seated during leisure time, participants who had more than six hours per day being seated during leisure time had an increased risk of CHD (HR, 1.26; 95% CI, 1.17–1.35), stroke (HR, 1.15; 95%CI, 1.03–1.28) and all CVD (HR, 1.19; 95%CI, 1.13–1.25).

## Discussion

The findings from this systematic review and meta-analysis, based on 14,51,730 participants from 17 prospective studies and two cross-sectional studies and including over 48,668 unique CVD cases and deaths, demonstrated that CVD was significantly associated with more than 10–11 h/d of total sedentary time (morbidity, RR, 1.24; 95% CI, 1.21–1.27; mortality, HR, 1.29; 95% CI, 1.13–1.47) which is similar with Pandey’s dose-response meta-analysis, and significantly associated with more than 5–6 h/d of screen time (morbidity, HR, 1.04; 95% CI, 1.03–1.05; mortality, HR, 1.36; 95% CI, 1.03–1.78) after adjustment for physical activity and other CVD risks. The differences between the highest and the lowest screen time categories in Cumming’s (2 vs 1.64 h/d) and Morales’s (2.5 vs 1 h/d) study were small, which potentially influenced the effect sizes. Thus, in sensitivity analysis, these two studies were excluded when assessing the association between screen time and CVD morbidity, and the association was further strengthened but became insignificant (HR, 1.22; 95% CI, 0.98–1.52; *P* = 0.07). The associations between occupational sitting time, leisure sedentary time, and CVD risks stayed inconclusive due to the small number or conflicting results of the included studies, which need more epidemiology studies to further confirm.

With the technological advances, we spend most of our leisure time being in front of electronic screens, such as television, computer, video, and smartphone. This study found that as a marker of total sedentary time, screen time had similar CVD risks with total sedentary time. Given that not all the included studies adjusted for total sedentary time or other types of sedentary time when assessing the association between screen time and CVD risks except for Garcia’s study, the results should be interpreted carefully. The exact mechanisms that explained the pathogenesis between sedentary time and CVD are still unclear and warrant further research, but several possible hypotheses have been proposed by clinical trials and epidemiological studies. The most persuasive hypothesis is that sedentary time increased the production and accumulation of reactive oxygen species, which is correlated with the increased cytokine release and other inflammatory markers, eventually leading to endothelial dysfunction [29–31]. There is evidence that the consumption of energy-dense, nutrient-poor snack foods increases during screen time, and a cross-sectional study from Australia in 2013 demonstrated that screen time and snack food consumption were jointly associated with the metabolic syndrome and its components, this may lead to a higher cardiovascular risk [32].

Our findings have important public health implications. Current guidelines emphasized the cardioprotective role of physical activity, in contrast, there is no guideline targeting sedentary behavior. Thus, it is unclear for adults how many hours per day of sedentary behavior is harmful and how to attenuate, even eliminate the risk caused by prolonged and uninterpreted sedentary behavior. Since adults spend most of their waking hours doing sedentary activities [13, 16], the lack of relevant guidelines is an important gap in the public health area. For adults who cannot tolerate more than 150 min of moderate physical activity or 75 min of vigorous physical activity per week, avoiding a high volume of sedentary time has particular importance. Sit-stand, treadmill, and bicycle workstations have been designed to interrupt prolonged occupational sitting time and proved as effective and beneficial interventions [33, 34]. However, interventions for adults with prolonged screen time out of work are limited and there is a need for evidence-based interventions to reduce leisure screen time, since that over 5–6 h/d of screen time may have similar CVD risks with over 10 h/d of total sedentary time.

This systematic review and meta-analysis have several limitations. First, the study protocol was not registered previously. Second, we only included English or Chinese language studies when literature screening, which may lead to publication bias and limit the generalizability of the study. Third, since other CVD risk factors are likely to influence the association between sedentary time and risk of CVD, the discrepancy of covariates included in the most adjusted models may potentially produce bias. Lastly, only three of the included studies measured sedentary time with the objective accelerometer, and many other included studies measured sedentary time with self-reported questions without reported reliability or validity, which may limit the objectivity of the results. However, the large sample sizes and long follow-up durations of our included studies may offset some of these limitations.

## Conclusion

This systematic review and meta-analysis demonstrated that a total sedentary time of more than 10–11 h/d and a screen time of more than 5–6 h/d had similar CVD risks after adjustment for physical activity and other CVD risk factors. Future studies are needed to explore the mechanism under sedentary time and CVD risks, and conduct evidence-based interventions to reduce sedentary time, especially screen time.

## Supplementary Information

**Additional file 1.**

**Additional file 2.**

## Data Availability

All data generated or analyzed during this study are included in this published article and its supplementary file 2.
