# Supplementary material for: Sedentary time and its association with risk of cardiovascular diseases in adults: an updated systematic review and meta-analysis of observational studies
Source: BMC Public Health. 2022 Feb 12;22:286. doi: 10.1186/s12889-022-12728-6 (PMC8840786; doi:10.1186/s12889-022-12728-6)
Supplement: Supplementary file 2 — Additional file 2. [file 12889_2022_12728_MOESM2_ESM.docx]

Table S1. Search terms of English databases

Pubmed

| #1 | "Sedentary Time"[MeSH Terms] OR "sedentary behaviour"[Title/Abstract] OR "sedentary lifestyle"[Title/Abstract] OR "sedentary time"[Title/Abstract] OR "sedent*"[Title/Abstract] OR "sit*"[Title/Abstract] OR "seated"[Title/Abstract] OR "Screen Time"[MeSH Terms] OR "Television"[MeSH Terms] OR "Computers"[MeSH Terms] OR "Video Games"[MeSH Terms] OR "televisions"[Title/Abstract] OR "television viewing"[Title/Abstract] OR "television watching"[Title/Abstract] OR "TV viewing"[Title/Abstract] OR "TV watching"[Title/Abstract] OR "television time"[Title/Abstract] OR "watch television"[Title/Abstract] OR "view television"[Title/Abstract] OR "watch TV"[Title/Abstract] OR "TV time"[Title/Abstract] OR "TV"[Title/Abstract] OR "screen behavior"[Title/Abstract] OR "screen behaviour"[Title/Abstract] OR "computer"[Title/Abstract] OR "video gaming"[Title/Abstract] OR "occupational sitting"[Title/Abstract] OR "work"[Title/Abstract] OR "transportation"[Title/Abstract] |
| --- | --- |
| #2 | "Coronary Disease"[MeSH Terms] OR "Stroke"[MeSH Terms] OR "Myocardial Infarction"[MeSH Terms] OR "Cerebral Arterial Diseases"[MeSH Terms] OR "Brain Infarction"[MeSH Terms] OR "Cardiovascular Diseases"[MeSH Terms] OR "CVD"[Title/Abstract] OR "cardiovascular disease"[Title/Abstract] OR "CHD"[Title/Abstract] OR "coronary heart disease"[Title/Abstract] OR "heart attack"[Title/Abstract] OR "myocardial infarct"[Title/Abstract] OR "brain infarct"[Title/Abstract] OR "coronary arteriosclerosis"[Title/Abstract] OR "MI"[Title/Abstract] |
| #3 | "Cross-Sectional Studies"[MeSH Terms] OR "Cohort Studies"[MeSH Terms] OR "Case-Control Studies"[MeSH Terms] OR "Mortality"[MeSH Terms] OR "Morbidity"[MeSH Terms] OR "Death"[MeSH Terms] OR "Risk Factors"[MeSH Terms] OR "crude death rate"[Title/Abstract] OR "crude mortality rate"[Title/Abstract] OR "death rate"[Title/Abstract] OR "incidence"[Title/Abstract] OR "risk"[Title/Abstract] |
| #4 | 80 and over[Filter] OR all adult[Filter] OR young adult[Filter] OR adult[Filter] OR middle aged aged[Filter] OR middle aged[Filter] OR aged[Filter] |
| #5 | "Randomized Controlled Trial"[Publication Type] OR "Review"[Publication Type] |
| #6 | "Child"[MeSH Terms] OR "Infant"[MeSH Terms] OR "Adolescent"[MeSH Terms] |
| #7 | #1 AND #2 AND #3 AND #4 NOT #5 NOT #6 |
| Limit: publication date from January 2015 to November 2021. | |
| Number of literature: 1962 | |

Cochrane Library

| #1 | MeSH descriptor: [Sedentary Time] explode all trees |
| --- | --- |
| #2 | MeSH descriptor: [Television] explode all trees |
| #3 | MeSH descriptor: [Computers] explode all trees |
| #4 | MeSH descriptor: [Video Games] explode all trees |
| #5 | MeSH descriptor: [Smartphone] explode all trees |
| #6 | MeSH descriptor: [Screen Time] explode all trees |
| #7 | ("Sedentary Time" or "sedentary lifestyle" or "sedentary time" or "sitting" or "sit" or "seated" or "screen behavior" or "televisions" or "television viewing" or "television watching" or "television time" or "TV" or "TV watching" or "TV viewing" or "watch television" or "view television" or "TV time" or "watch TV" or "computer" or "video gaming" or "occupational sitting" or "work" or "transportation"):ti,ab,kw |
| #8 | MeSH descriptor: [Cardiovascular Diseases] explode all trees |
| #9 | MeSH descriptor: [Coronary Disease] explode all trees |
| #10 | MeSH descriptor: [Stroke] explode all trees |
| #11 | MeSH descriptor: [Myocardial Infarction] explode all trees |
| #12 | MeSH descriptor: [Cerebral Arterial Diseases] explode all trees |
| #13 | MeSH descriptor: [Brain Infarction] explode all trees |
| #14 | ("CVD" OR "cardiovascular disease" OR "CHD" OR "coronary heart disease" OR "heart attack" OR "myocardial infarct" OR "brain infarct" OR "coronary arteriosclerosis" OR "MI"):ti,ab,kw |
| #15 | MeSH descriptor: [Cross-Sectional Studies] explode all trees |
| #16 | MeSH descriptor: [Cohort Studies] explode all trees |
| #17 | MeSH descriptor: [Case-Control Studies] explode all trees |
| #18 | MeSH descriptor: [Mortality] explode all trees |
| #19 | MeSH descriptor: [Death] explode all trees |
| #20 | MeSH descriptor: [Risk Factors] explode all trees |
| #21 | ("crude death rate" OR "crude mortality rate" OR "death rate" OR "incidence" OR "risk"):ti,ab,kw |
| #22 | (#1 OR #2 OR #3 OR #4 OR #5 OR #6 OR #7) AND (#8 OR #9 OR #10 OR #11 OR #12 OR #13 OR #14) AND (#15 OR #16 OR #17 OR #18 OR #19 OR #20 OR #21) |
| Limit: publication date from January 2015 to November 2021. | |
| Number of literature: 2351 | |

Web of Science

| #1 | AB=("Sedentary Time" or "sedentary lifestyle" or "sedentary time" or "sitting" or "sit" or "seated" or "screen behavior" or "television" or "televisions" or "television viewing" or "television watching" or "television time" or "TV" or "TV watching" or "TV viewing" or "watch television" or "view television" or "TV time" or "watch TV" or "computers" or "computer" or "video game" or "video gaming" or "occupational sitting" or "work" or "transportation") |
| --- | --- |
| #2 | AB=("Cardiovascular Diseases" OR "CVD" OR "cardiovascular disease" OR "Coronary Disease" OR "CHD" OR "coronary heart disease" OR "heart attack" OR "myocardial infarction" OR "myocardial infarct" OR "Cerebral Arterial Diseases" OR "Brain Infarction" OR "brain infarct" OR "coronary arteriosclerosis" OR "MI") |
| #3 | AB=("Cross-sectional Studies" OR "Cohort Studies" OR "Case-control Studies" OR "Mortality" OR "Morbidity" OR "death" OR "crude death rate" OR "crude mortality rate" OR "death rate" OR "incidence" OR "risk factors" OR "risk") |
| #4 | #1 AND #2 AND #3 |
| Limit: publication date from January 2015 to November 2021. | |
| Number of literature: 1405 | |

Embase

| #1 | 'Sedentary Time':ab,ti OR 'sedentary lifestyle':ab,ti OR 'sedentary time':ab,ti OR 'sitting':ab,ti OR 'sit':ab,ti OR 'seated':ab,ti OR 'television':ab,ti OR 'television viewing':ab,ti OR 'television watching':ab,ti OR 'television time':ab,ti OR 'televisions':ab,ti OR 'watch television':ab,ti OR 'view television':ab,ti OR 'TV watching':ab,ti OR 'TV viewing':ab,ti OR 'watch TV':ab,ti OR 'TV time':ab,ti OR 'computers':ab,ti OR 'computer':ab,ti OR 'video games':ab,ti or 'video gaming':ab,ti or 'smartphone':ab,ti or 'occupational sitting':ab,ti or 'work':ab,ti or 'transportation':ab,ti |
| --- | --- |
| #2 | 'cardiovascular diseases':ab,ti OR 'cardiovascular disease':ab,ti OR 'cvd':ab,ti OR 'coronary disease':ab,ti OR 'coronary heart disease':ab,ti OR 'chd':ab,ti OR 'stroke':ab,ti OR 'myocardial infarction':ab,ti OR 'myocardial infarct':ab,ti OR 'mi':ab,ti OR 'brain infarction':ab,ti OR 'cerebral arterial diseases':ab,ti OR 'coronary arteriosclerosis':ab,ti OR 'heart attack':ab,ti |
| #3 | 'cross-sectional study':ab,ti OR 'cohort study':ab,ti OR 'case-control study':ab,ti OR 'mortality':ab,ti OR 'morbidity':ab,ti OR 'death':ab,ti OR 'incidence':ab,ti OR 'risk factors':ab,ti OR 'risk':ab,ti OR 'crude mortality rate':ab,ti OR 'crude death rate':ab,ti OR 'death rate':ab,ti |
| #4 | (2015:py OR 2016:py OR 2017:py OR 2018:py OR 2019:py OR 2020:py OR 2021:py) AND 'human'/de AND 'article'/it AND ([adult]/lim OR [aged]/lim OR [middle aged]/lim OR [very elderly]/lim OR [young adult]/lim) |
| #5 | #1 AND #2 AND #3 AND #4 |
| Limit: publication date from January 2015 to November 2021. | |
| Number of literature: 1033 | |

Table S2. Reported Average Duration/Time Range and Estimated Median Duration (in hours/day) for Total Sedentary Time and Screen Time Categories Across Included Studies

| Publication | Type of Sedentary Time | Reported Time and Estimated Median | Reference Category, h/d | Category 2, h/d | Category 3, h/d | Category 4, h/d | | | Category 5, h/d | Category 6, h/d | | Category 7, h/d | |
| --- | --- | --- | --- | --- | --- | --- | --- | --- | --- | --- | --- | --- | --- |
| Park, 2021 | Total sedentary time | Reported Time | 0-6 | ≥7 | NA | | | | | | | | |
|  |  | Estimated Median | **3** | **10** |  |  |  |  |  |  |  |  |  |
| Hamer, 2020 | Screen time | Reported Time | 1 | 2 | NA | | | | | | | | |
|  |  | Estimated Median | **1** | **2** |  |  |  |  |  |  |  |  |  |
| Liu, 2020 | Total sedentary time | Reported Time | ＜5 | 5-＜8 | 8-＜10 | ≥10 | | | NA | | | | |
|  |  | Estimated Median | **2.5** | **6.5** | **9** | **11** | | |  |  |  |  |  |
| Tu, 2020 | Total sedentary time | Reported Time | ＜2 | ≥4 | NA | | | | | | | | |
|  |  | Estimated Median | **1** | **5** |  |  |  |  |  |  |  |  |  |
| Bellettiere, 2019 | Total sedentary time | Reported Time | 3.28-8.18 | 8.20-9.18 | 9.20-10.20 | 10.22-14.08 | | | NA | | | | |
|  |  | Estimated Median | **5.73** | **8.69** | **9.70** | **12.15** | | |  |  |  |  |  |
| Garcia, 2019 | Screen time | Reported Time | ＜2 | 2-4 | ＞4 | NA | | | | | | | |
|  |  | Estimated Median | **1** | **3** | **5** |  |  |  |  |  |  |  |  |
| Stamatakis, 2019 | Total sitting time | Reported Time | ＜4 | 4-＜6 | 6-≤8 | ＞8 | NA | | | | | | |
|  |  | Estimated Median | **2** | **5** | **7** | **9** |  |  |  |  |  |  |  |
| Morales, 2018 | Screen time | Reported Time | ＜2 | 2-3 | NA | | | | | | | | |
|  |  | Estimated Median | **1** | **2.5** |  |  |  |  |  |  |  |  |  |
| Dohrn, 2017 | Total sedentary time | Reported Time | 6.55±0.82 | 8.20±0.36 | 9.83±0.83 | NA | | | | | | | |
|  |  | Estimated Median | **6.55** | **8.20** | **9.83** |  |  |  |  |  |  |  |  |
| Engelen, 2017 | Total sitting time | Reported Time | ＜5.14 | ＞5.14 | NA | | | | | | | | |
|  |  | Estimated Median | **2.57** | **7.71** |  |  |  |  |  |  |  |  |  |
| Cumming, 2016 | Screen time | Reported Time | 1.64 | 2 | NA | | | | | | | | |
|  |  | Estimated Median | **1.64** | **2** |  |  |  |  |  |  |  |  |  |
| Evenson, 2016 | Total sedentary time | Reported Time | ＜6.89 | 6.89-8.29 | 8.30-9.80 | ≥9.81 | | NA | | | | | |
|  |  | Estimated Median | **3.45** | **7.59** | **9.05** | **10.56** | |  |  |  |  |  |  |
|  | Screen time | Reported Time | ≤2 | 2.1-3.5 | ≥3.6 | NA | | | | | | | |
|  |  | Estimated Median | **1** | **2.8** | **4.3** |  |  |  |  |  |  |  |  |
| Grace, 2016 | Screen time | Reported Time | ＜2 | 2-＜4 | ≥4 | NA | | | | | | | |
|  |  | Estimated Median | **1** | **3** | **5** |  |  |  |  |  |  |  |  |
| McDonnell, 2016 | Screen time | Reported Time | ＜2 | 2-＜4 | ≥4 | NA | | | | | | | |
|  |  | Estimated Median | **1** | **3** | **5** |  |  |  |  |  |  |  |  |
| Moller, 2016 | Occupational sitting time | Reported Time | ＜3.57 | ≥3.57 | NA | | | | | | | | |
|  |  | Estimated Median | **1.79** | **5.36** |  |  |  |  |  |  |  |  |  |
| Borodulin, 2015 | Total sedentary time | Reported Time | 1 | 2 | NA | | | | | | | | |
|  |  | Estimated Median | **1** | **2** |  |  |  |  |  |  |  |  |  |
| Ikehara, 2015 | Screen time | Reported Time | ＜2 | 2 | 3 | 4 | | 5 | | | 6 | | ≥6 |
|  |  | Estimated Median | **1** | **2** | **3** | **4** | | **5** | | | **6** | | **7** |
| Keadle, 2015 | Screen time | Reported Time | ＜1 | 1-2 | 3-4 | 5-6 | | ＞7 | | | NA | | |
|  |  | Estimated Median | **0.5** | **1.5** | **3.5** | **5.5** | | **7.5** | | |  |  |  |

Abbreviations: NA: non-available

Table S3a. Sedentary Time Categories and Associated Risk for CVD Morbidity Across Included Studies

| Publication | Categories of Sedentary Time | Most adjusted HR/RR by categories (95% CI) | Covariates in the most adjusted model |
| --- | --- | --- | --- |
| Park, 2021 | **Total sedentary time**, h/d  1: 0-6  2: ≥7 | Morbidity of **CVD**  OR  1: 1.00  2: 1.28 [1.08, 1.51]  RR (after corrected)  1: 1.00  2: 1.24 [1.21, 1.27] | Sex, age, education, household income, marital status, **total PA**, waist circumference, current smoking, high-risk alcohol consumption, chronic diseases (diabetes mellitus, hypertension, dyslipidemia), and self-rated health |
| Liu, 2020 | **Total sedentary time**, h/d  1: ＜5  2: 5-＜8  3: 8-＜10  4: ≥10 | Morbidity of **stroke**, HR  1: 1.00  2: 1.00 [0.87, 1.14]  3: 1.11 [0.96, 1.28]  4: 1.19 [1.03, 1.38] | Sex, age, geographic region, urbanization, education, family history of CVD, current smoking status, alcohol consumption and **MVPA** |
|  |  | Morbidity of **CHD**, HR  1: 1.00  2: 1.08 [0.84, 1.41]  3: 1.52 [1.17, 1.98]  4: 2.12 [1.63, 2.75] |  |
| Garcia, 2019 | **Screen time**, h/d  1: ＜2  2: 2-4  3: ＞4 | Morbidity of **CVD**, HR  1: 1.00  2: 1.10 [0.73, 1.66]  3: 1.43 [0.96, 2.12] | Age, sex, education, alcohol consumption, smoking status, health diet, body mass index, hypertension, diabetes mellitus, history of myocardial infarction/stroke, **leisure time** **MVPA**, occupational sitting |
|  | **Occupational sitting time**  1: never or seldom  2: sometimes  3: often or always | Morbidity of **CVD**, HR  1: 1.00  2:1.01 [0.67, 1.51]  3: 1.06 [0.73, 1.55] | Age, sex, education, alcohol consumption, smoking status, health diet, body mass index, hypertension, diabetes mellitus, history of myocardial infarction/stroke, **leisure time** **MVPA,** screen time |
| Morales, 2018 | **Screen time**, h/d  1: ＜2  2: 2-3 | Morbidity of **CVD**, HR  1: 1.00  2: 1.04 [1.03, 1.05] | **Walking and MVPA**, grip strength, categories of sleep duration, dietary intake (alcohol, fruit and vegetables, red meat, processed meat and oily fish intake), systolic blood pressure, prevalent diabetes, hypertension and medication for diabetes, hypertension, and cholesterol. Ex-smokers and current smokers were excluded. |
| Engelen, 2017 | **Total sitting time**, h/week  1: ＜36  2: ＞36 | Morbidity of **CVD**, OR  1: 1.00  2: 1.28 [1.02-1.60] | Waist circumference, occupation, age, sex, self-rated health, smoking status, **total PA** |
| Cumming, 2017 | **Screen time**, h/d  1: 1.64  2: 2 | Morbidity of **Stroke**, HR  1: 1.00  2: 1.00 [0.997, 1.002] | Age, sex, education,smoking status, alcohol intake, diet quality |
| McDonnell, 2016 | **Screen time**, h/d  1: ＜2  2: 2-＜4  3: ≥4 | Morbidity of **stroke**, HR  1: 1.00  2: 1.10 [0.87, 1.40]  3: 1.14 [0.88, 1.48] | Age, sex, race, region, age-race interaction, **MVPA**, general health, marital status, employment status, depressive symptoms |

Abbreviations: CVD: Cardiovascular Disease; CHD: Coronary Heart Disease; MVPA: Moderate-to-Vigorous Physical Activity; PA: Physical Activity

Table S3b. Sedentary Time Categories and Associated Risk for CVD Mortality Across Included Studies

| Publication | Categories of Sedentary Time | Most adjusted HR by categories (95% CI) | Covariates in the most adjusted model |
| --- | --- | --- | --- |
| Hamer, 2020 | **Screen time**, h/d  1: ＜1  2: each additional hour | Mortality of **IHD**, HR  1: 1.00  2: 1.09 [1.06, 1.12] | Age, sex, smoking, education and prevalent CVD (angina, heart attack, hypertension) |
| Liu, 2020 | **Total sedentary time**, h/d  1: ＜5  2: 5-＜8  3: 8-＜10  4: ≥10 | Mortality of **CVD**, HR  1: 1.00  2: 0.94 [0.79, 1.13]  3: 1.00 [0.82, 1.21]  4: 1.27 [1.05, 1.52] | Sex, age, geographic region, urbanization, education, family history of CVD, current smoking status, alcohol consumption and **MVPA** |
| Stamatakis, 2019 | **Total sitting time**, h/d  1: ＜4  2: 4-＜6  3: 6-≤8  4: ＞8 | Mortality of **CVD**, HR  1: 1.00  2: 1.13 [0.98, 1.31]  3: 1.23 [1.06, 1.41]  4: 1.26 [1.04, 1.54] | Age, sex, education, marital status, remoteness, body mass index, smoking, self-rated health, total fruit and vegetable consumption, help for disability, psychological distress, diabetes, and **MVPA** |
| Patel, 2018 | **Leisure sedentary time**, h/d  1: ＜3  2: 3-5  3: ≥6 | Mortality of **CHD**, HR  1: 1.00  2: 1.07 [1.02, 1.13]  3: 1.26 [1.17, 1.35] | Sex, race, education, employment status, alcohol intake, marital status, smoking status, comorbidities, American Cancer Society Diet Score, aspirin use, body mass index, **recreational MVPA** |
|  |  | Mortality of **Stroke**, HR  1: 1.00  2: 1.04 [0.96, 1.12]  3: 1.15 [1.03, 1.28] |  |
|  |  | Mortality of **total CVD**, HR  1: 1.00  2: 1.06 [1.02, 1.10]  3: 1.19 [1.13, 1.25] |  |
| Dohrn, 2017 | **Total sedentary time**, min/d  1: 392.8±49.1  2: 491.9±21.4  3: 590.0±49.6 | Mortality of **CVD**, HR  1: 1.00  2: 1.63 [0.36,7.28]  3: 5.51 [1.43, 21.23] | Age, sex, education, smoking, health conditions at baseline (hypertension, heart disease, cancer, diabetes), achieving at least 150 min of **MVPA** per week and **accelerometer wear time**. |
| Evenson, 2016 | **Total sedentary time**, min/d  1: ≤413.4  2: 413.5-497.6  3: 497.7-588.3  4: ≥588.4 | Mortality of **CVD**, HR  1: 1.00  2: 1.12 [0.54-2.31]  3: 1.03 [0.50-2.12]  4: 1.44 [0.71-2.90] | **Average daily accelerometer wear time**, age, sex, race/ethnicity, education, married, cigarette smoking, employment, need special equipment to walk, arthritis, cancer, body mass index, hypertension, diabetes, **total PA** |
|  | **Screen time**, h/d  1: ≤2  2: 2.1-3.5  3: ≥3.6 | Mortality of **CVD**, HR  1: 1.00  2: 0.85 [0.51, 1.42]  3: 0.90 [0.57, 1.41] |  |
| Grace, 2016 | **Screen time**, h/d  1: ＜2  2: 2-＜4  3: ≥4 | Mortality of **CVD** in non-smokers, HR  1: 1.00  2: 0.93 [0.69, 1.26]  3: 1.04 [0.69, 1.57] | Age, sex, **leisure time PA**, education, household income, total energy intake, alcohol intake, Dietary Guideline Index, hypertension or anti-hypertensive medication use, total plasma cholesterol,  HDL-C, serum triglycerides, lipid-lowering medication use, and glucose tolerance status. |
|  |  | Mortality of **CVD** in current-smokers, HR  1: 1.00  2: 1.11 [0.46, 2.63]  3: 2.02 [0.80, 5.12] |  |
| Ikehara, 2015 | **Screen time**, h/d  1: ＜2  2: =2  3: =3  4: =4  5: =5  6: ≥6 | Mortality of **stroke**, HR  1: 1.00  2: 1.06 [0.93, 1.19]  3: 0.98 [0.87, 1.11]  4: 0.89 [0.76, 1.03]  5: 1.06 [0.90, 1.23]  6: 1.10 [0.92, 1.32] | Age, sex, body mass index, smoking, ethanol intake, education, **hours of sport, hours of walking**, sleep duration, perceived mental stress, presence of job, frequency of fresh fish intake, depressive symptoms, histories of hypertension and diabetes. |
|  |  | Mortality of **CAD**, HR  1: 1.00  2: 1.01 [0.84, 1.21]  3: 0.92 [0.77, 1.11]  4: 1.00 [0.81, 1.24]  5: 1.16 [0.92, 1.45]  6: 1.24 [0.96, 1.61] |  |
|  |  | Mortality of **total CVD**, HR  1: 1.00  2: 1.00 [0.92, 1.08]  3: 0.95 [0.88, 1.04]  4: 0.96 [0.87, 1.05]  5: 1.03 [0.93, 1.14]  6: 1.14 [1.02, 1.28] |  |
| Keadle, 2015 | **Screen time**, h/d  1: ＜1  2: 1-2  3: 3-4  4: 5-6  5: ＞7 | Mortality of **CVD**, HR  1: 1.00  2: 1.09 [0.97, 1.23]  3: 1.19 [1.06, 1.33]  4: 1.26 [1.11, 1.43]  5: 1.64 [1.42, 1.90] | Age, sex, race, education, smoking history, diet quality, **MVPA**, body mass index, self-reported health status |

Abbreviations: CVD: Cardiovascular Disease; IHD: Ischemic Heart Disease; MVPA: Moderate-to-Vigorous Physical Activity; CHD: Coronary Heart Diseases; PA: Physical Activity; CAD: Coronary Artery Diseases

Table S3c. Sedentary Time Categories and Associated Risk for CVD Morbidity and Mortality Across Included Studies

| Publication | Categories of Sedentary Time | Most adjusted HR by categories (95% CI) | Covariates in the most adjusted model |
| --- | --- | --- | --- |
| Tu, 2020 | **Total sedentary time**, h/d  1: ＜2  2: ≥4 | **Morbidity and mortality of CVD**, HR  1: 1.00  2: 2.07 [1.07-3.40] | Age, sex, education, marital status, region, smoking status, alcohol consumption, **total** **PA** and history of hypertension/ diabetes/ hyperlipidemia/ central obesity. |
| Bellettiere, 2019 | **Total sedentary time**, min/d  1: 197-491  2: 492-551  3: 552-612  4: 613-845 | **Morbidity and mortality of CVD**, HR  1: 1.00  2: 1.35 [0.97, 1.86]  3: 1.19 [0.84, 1.68]  4: 1.32 [0.91, 1.90] | Age, ethnicity, education, self-reported health status, family history of MI, multimorbidity, physical functioning, alcohol consumption, current smoking status, **MVPA**, **awake wear time of accelerometer** |
| Moller, 2016 | **Occupational sitting time**, h/week  1: ＜25  2: ≥25 | **Morbidity and mortality of IHD**, HR  1: 1.00  2: 0.94 [0.71-1.27] | Sex, age, socioeconomic status, smoking status, alcohol consumption, **leisure time PA**, decision latitude and calendar year. |
| Borodulin, 2015 | **Total sedentary time**, h/d  1: ＜1  2: each additional hour | **Morbidity and mortality of CVD**, HR  1: 1.00  2: 1.06 [1.01-1.11] | Age, sex, daily total sitting (continuous hours), employment status, education, smoking status, **leisure time PA**, body mass index, use of vegetables and fruit, alcohol consumption, high blood pressure and/or its medication, and high serum cholesterol and/or its medication. |

Abbreviations: CVD: Cardiovascular Disease; PA: Physical Activity; MVPA: Moderate-to-Vigorous Physical Activity; IHD: Ischemic Heart Disease

Table S4. Definitions and Measurements of Sedentary Time, Physical Activity (if included in the most adjusted models) and CVD Outcomes

| Publication | Definition of Sedentary Time | Measurement of Sedentary Time | Definition of PA | Measurement of PA | PA adjusted for in the most adjusted model | Definition of CVD Outcomes | Measurement of CVD Outcomes |
| --- | --- | --- | --- | --- | --- | --- | --- |
| Park, 2021 | Total sedentary time | **Self-reported** question: “How much time do you spend **sitting of lying down** a day?” | Total PA | **Global Physical Activity Questionnaire** Korean Version | Physical active or inactive | Morbidity of stroke, MI and angina | Self-reported |
| Hamer, 2020 | Screen time: television watching | **Self-reported** question: “In a typical day, how many hours do you spend **watching TV**? If the time you spend watching TV varies a lot, give the average time for a 24 hour day in the last 4 weeks”. | **PA was not included in the most adjusted model** | | | Mortality of IHD (ICD-10 I20-I25) | Death certifications |
| Liu, 2020 | Total sedentary time | **Self-reported** question: “During the previous year, how many hours in each  24-h day did you usually spend on the following activities (light, moderate, vigorous physical activity, **Sedentary Time** and sleep) on weekdays/weekends”. | MVPA | **Self-reported** question: “During the previous year, how many hours in each 24-h day did you usually spend on the following activities (light, **moderate, vigorous physical activity**, Sedentary Time and sleep) on weekdays/weekends”. | MVPA: ≥150 or ＜150 min/week | The first diagnosis of stroke (ICD-10 I60-I69), CHD (ICD-10 I20-I25), or CVD death (ICD-10 I00-I99) | Medical records and death certifications |
| Tu, 2020 | Total sedentary time | Self-reported question | Total PA | **Self-reported** question | High, moderate or low amount of total PA according to MET/min/week | CVD events: CHD, stroke and CVD death | Medical records |
| Bellettiere, 2019 | Total sedentary time | ActiGraph GT3X+ **accelerometer**: <100 counts/minute was defined as **Sedentary Time**. Participants who had ≥ 10 waking wear hours on ≥ 4 days per week were included. | MVPA | ActiGraph GT3X+ **accelerometer**: ≥519 vector magnitude accelerometer counts/15-seconds was defined as moderate-to-vigorous physical activity. | Median time spent doing MVPA per week | CVD events: the first occurrence of an MI, revascularization, hospitalized angina, heart failure, stroke, or death from any CVD. | Medical records and death certifications |
| Garcia, 2019 | Screen time (television watching) | **Self-reported** question: “During the past year, how often did you **watch television**?” | Leisure time MVPA | **Self-reported** question: about the yearly frequency and weekly duration of participation in exercise and sports over the past year. | Poor, intermediate or ideal leisure time MVPA (≥3.5 MET/h/week） | Morbidity of CHD or stroke | Medical records |
|  | Occupational sitting time | **Self-reported** question: “When you are **at work**, how often do you **sit**?” |  |  |  |  |  |
| Stamatakis, 2019 | Total sitting time | **Self-reported** question: “How many hours in each 24-h day do you usually spend doing the following? **Sitting**, standing and sleeping.” And in line with the **sitting questions** in the **International Physical Activity Questionnaire**. | MVPA | **Active Australia Survey questions**: “If you add up all the time you spent doing each activity last week, how much time did you spend altogether doing each type of activity?” | No physical activity, ≤149/150-299/300-419/≥420 min/week MVPA (≥3.5 MET/h/week） | CVD-related death | Death certifications |
| Patel, 2018 | Leisure sedentary time | **Self-reported** question: “During the past year, on an average day (**not counting time spent at your job**), how many hours per day did you **spend sitting** (watching television, reading, etc.)?” | Recreational MVPA | **Self-reported** question: “During the past year, what was the average time per week you spent at the following kinds of activities: walking, jogging/running, lap swimming, tennis or racquetball, bicycling or stationary biking, aerobics/calisthenics, and dancing?” | MVPA dose in MET/h/week | CVD mortality: CHD (ICD-9 410-414; ICD-10 I20-I25), stroke (ICD-9 430-438; ICD-10 I60-I69), and total CVD (ICD-9 390-459; ICD-10 I00-I99) | Death certifications |
| Morals, 2018 | Screen time (television viewing and personal computer using) | **Self-reported** question: “In a typical day, how many hours do you **spend watching TV**?”, “In a typical day, how many hours do you **spend using the computer**? (**Do not include using a computer at work**).” | Walking and MVPA | **International Physical Activity Questionnaire** short form. | Walking and MVPA dose in MET/h/week | CVD morbidity: ICD-10 I05-I89.9 | Medical records |
| Dohrn, 2017 | Total sedentary time | Actigraph 7164 **accelerometer:** <100 counts/minute was defined as Sedentary Time. Participants who had ≥10 waking wear hours on ≥1 days per week were included. | MVPA | Actigraph 7164 **accelerometer:** light intensity as 100–2019 counts/min, and MVPA as ≥2020 counts/min. | ≥150 min/week MVPA | CVD mortality | Death certifications |
| Engelen, 2017 | Total sitting time | **Self-reported** question: “how much time did you spend **sitting at work** in the last week?”, “In the last week did you spend time **sitting to travel to or from places**?” and “ how much time, **sitting or lying down to leisure activities**: 1. Watch television or videos; 2. Play electronic games; 3. Use a computer or the Internet; 4. Use a phone (e.g. text and talk); 5. Other social or leisure activities and; 6. None of these.” | Total PA | **Active Australia Questionnaire**: total PA was calculated as the sum of the reported minutes spent walking, doing MPA, and twice the total minutes spent doing VPA. | Quartiles 3-4 of total PA | CVD morbidity | Self-reported |
| Cumming, 2016 | Screen time (television watching or video) | **Self-reported** question | **PA was not included in the most adjusted model** | |  | Morbidity of stroke | Medical records |
| Evenson, 2016 | Total sedentary time | Actigraph 7164 **accelerometer:** <100 counts/minute was defined as Sedentary Time. Participants who had ≥8 waking wear hours on ≥3 days per week were included. | Total PA | Actigraph 7164 **accelerometer**: light intensity as 100–2019 counts/min, and MVPA as ≥2020 counts/min. | Total PA dose in MET/h/week (quartiles) | CVD mortality | Death certifications |
|  | Screen time | **Self-reported** question |  |  |  |  |  |
| Grace, 2016 | Screen time (television viewing or video) | **Self-reported** question | Leisure time PA | **Active Australia questionnaire** | Average time spent doing leisure time PA per day | CVD mortality (ICD-10  I10–I25, I46.1, I48, I50–I99 or R96) | Death certifications |
| McDonnell, 2016 | Screen time (television watching or video) | **Self-reported** question: “How many hours do you **watch television or video**, per day  or per week, on average?” | MVPA | **Self-reported** question: number of times per week engaged in intense physical activity, sufficient to  work up a sweat. | Number of times per week engaged in MVPA | CVD morbidity: stroke | Medical records |
| Moller, 2016 | Occupational sitting time | **Self-reported** question: “Does your **job** involve **sitting**?” | Leisure time PA | **Self-reported** question | ≥2/＜2 h/week | Morbidity and mortality of IHD (ICD-8 410-414, ICD I20-I25) | Medical records and death certifications |
| Borodulin, 2015 | Total sedentary time | **Self-reported** question: “How much time did you spend **sitting** on a typical weekday? This includes **work and leisure time sitting**, sitting at home and while visiting, and studying and travelling. This also includes sitting or lying down while reading or watching TV.” | Leisure time PA | **Self-reported** question: “How much do you exercise and stress yourself  physically in your leisure time?” | Physical active or inactive | CVD morbidity and mortality: fatal cases of IHD (ICD-10 I20-I25, I46, R96, R98), nonfatal cases of IHD (ICD-10 I20-I25), and fatal and nonfatal strokes (ICD-10 I61, I63 (not I636), I64) | Medical records and death certifications |
| Ikehara, 2015 | Screen time (television watching) | **Self-reported** question: “On average how many hours do you **watch TV**?” | Sport and walking | Self-reported question | Average time spent doing sports or walking per week or per day | Mortality of stroke (ICD-10 I60-I69), CAD (I20-I25) and total CVD (I01-I99). | Death certifications |
| Keadle, 2015 | Screen time (television watching and video) | **Self-reported** question: “During a typical 24-hour period over the past 12 months, how much time did you **spend watching television or videos**?” | MVPA | Self-reported question | Average time spent doing MVPA per week | Mortality of disease of the heart | Death certifications |

Abbreviations: CVD: Cardiovascular Diseases; PA: Physical Activity; MVPA: Moderate-to-Vigorous Physical Activity; ICD: International Classification of Diseases; MI: Myocardial Infarction; IHD: Ischemic Heart Disease; CHD: Coronary Heart Disease; MPA: Moderate Physical Activity; VPA: Vigorous Physical Activity; CAD: Coronary Artery Disease

**Table S5. Quality assessment scores of the included studies**

| Publication | 1 point for prospective study design | If sedentary time was self-reported, 1 for reported reliability, 1 for reported validity. | 2 If an objective measure of sedentary time was used. | 1 if two or more covariates were controlled for | 1 if analysis controlled for physical activity | 1 for an objective measure of the health outcome | Total score |
| --- | --- | --- | --- | --- | --- | --- | --- |
| Park, 2021 | 0 | 1 | - | 1 | 1 | 0 | 3 |
| Harmer, 2020 | 1 | 0 | - | 1 | 0 | 1 | 3 |
| Liu, 2020 | 1 | 0 | - | 1 | 1 | 1 | 4 |
| Tu, 2020 | 1 | 0 | - | 1 | 1 | 1 | 4 |
| Bellettiere, 2019 | 1 | - | 2 | 1 | 1 | 1 | 6 |
| Garcia, 2019 | 1 | 2 | - | 1 | 1 | 1 | 6 |
| Stamatakis, 2019 | 1 | 1 | - | 1 | 1 | 1 | 5 |
| Patel, 2018 | 1 | 0 | - | 1 | 1 | 1 | 4 |
| Morales, 2018 | 1 | 0 | - | 1 | 1 | 1 | 4 |
| Dohrn, 2017 | 1 | 0 | 2 | 1 | 1 | 1 | 6 |
| Engelen, 2016 | 0 | 2 | - | 1 | 1 | 1 | 5 |
| Cumming, 2016 | 1 | 1 | - | 1 | 0 | 1 | 4 |
| Evenson, 2016 | 1 | - | 2 | 1 | 1 | 1 | 6 |
| Grace, 2016 | 1 | 2 | - | 1 | 1 | 1 | 6 |
| McDonnel, 2016 | 1 | 2 | - | 1 | 1 | 1 | 6 |
| Moller, 2016 | 1 | 0 | - | 1 | 1 | 1 | 4 |
| Borodulin, 2015 | 1 | 2 | - | 1 | 1 | 1 | 6 |
| Ikehara, 2015 | 1 | 0 | - | 1 | 1 | 1 | 4 |
| Keadle, 2015 | 1 | 1 | - | 1 | 1 | 1 | 5 |


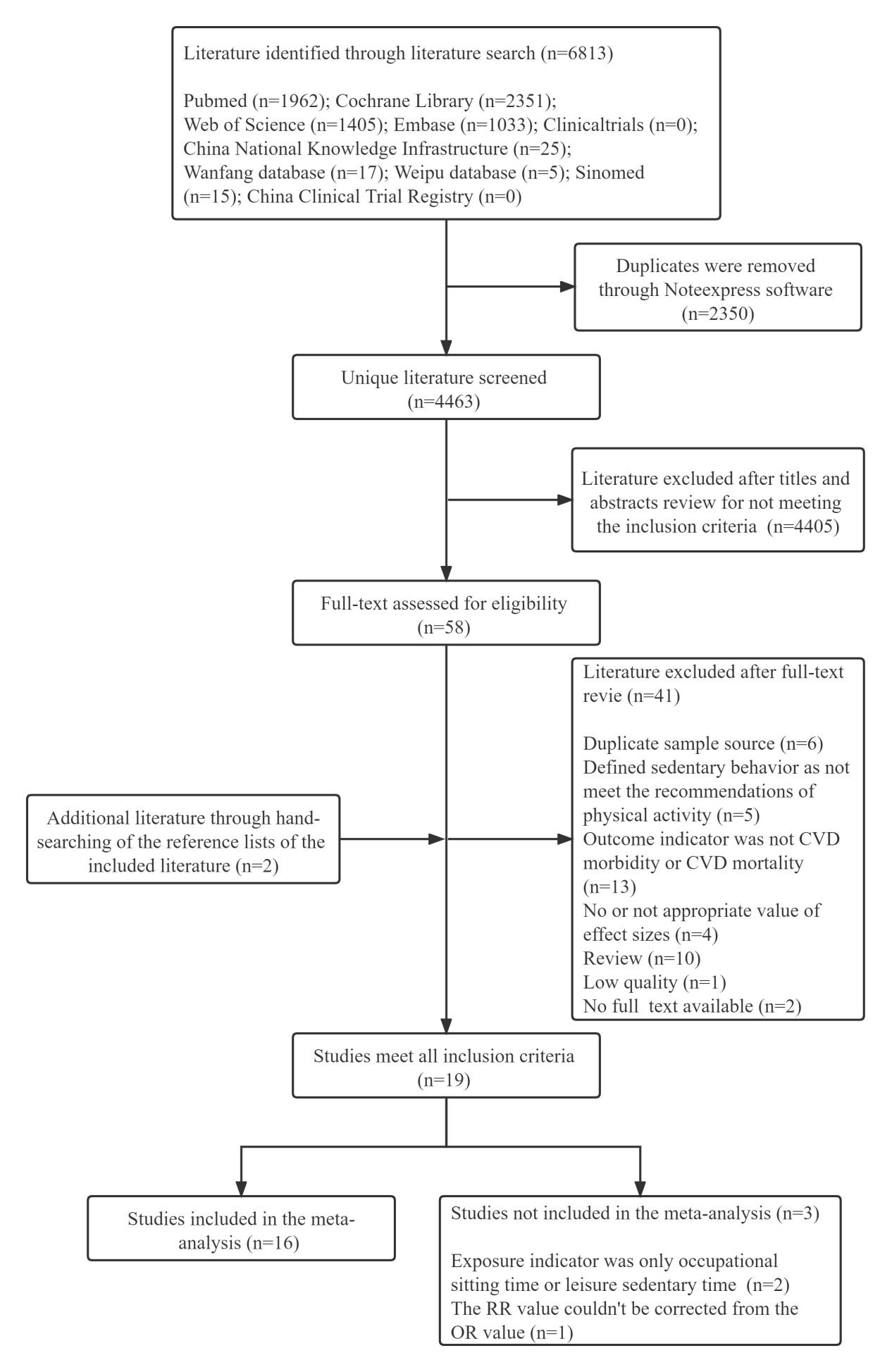


Figure S1. The flowchart of the literature search


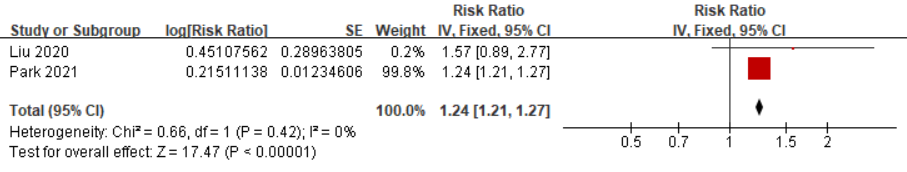


Figure S2. Forest Plot of the Association Between Highest versus Lowest Total Sedentary Time Duration And CVD Morbidity


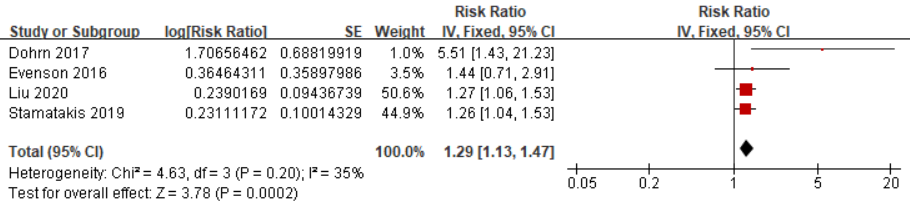


Figure S3. Forest Plot of the Association Between Highest versus Lowest Total Sedentary Time Duration And CVD Mortality


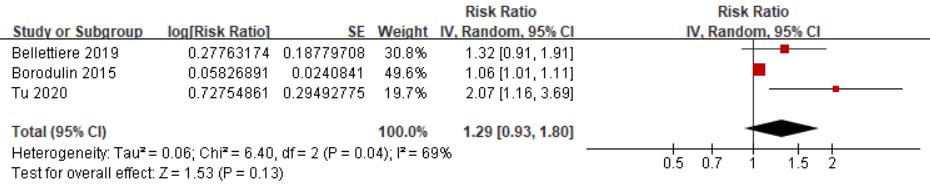


Figure S4. Forest Plot of the Association Between Highest versus Lowest Total Sedentary Time Duration And CVD Morbidity And Mortality


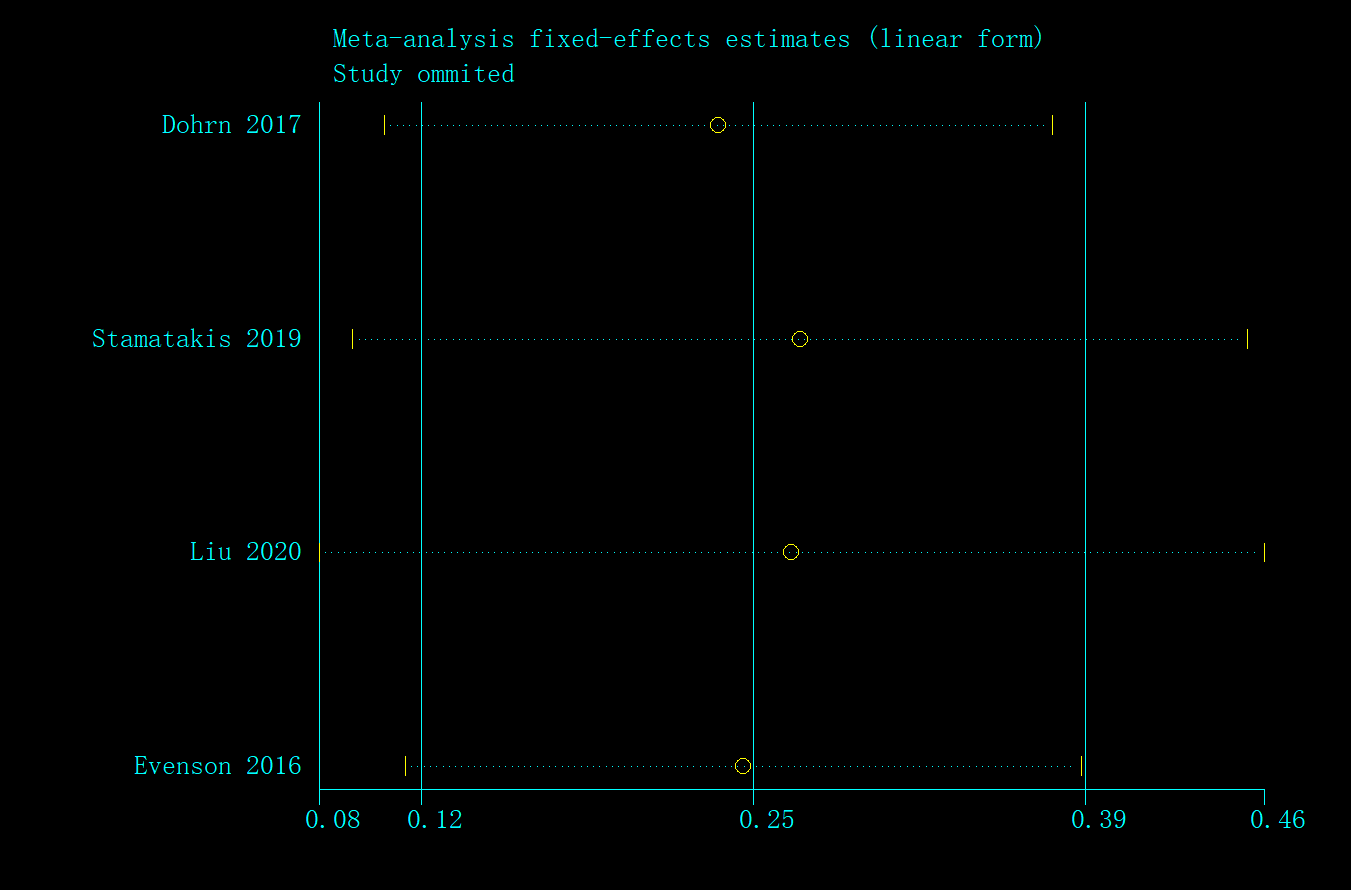


Figure S5. Sensitivity Analysis of the Association Between Highest versus Lowest Total Sedentary Time And CVD Mortality


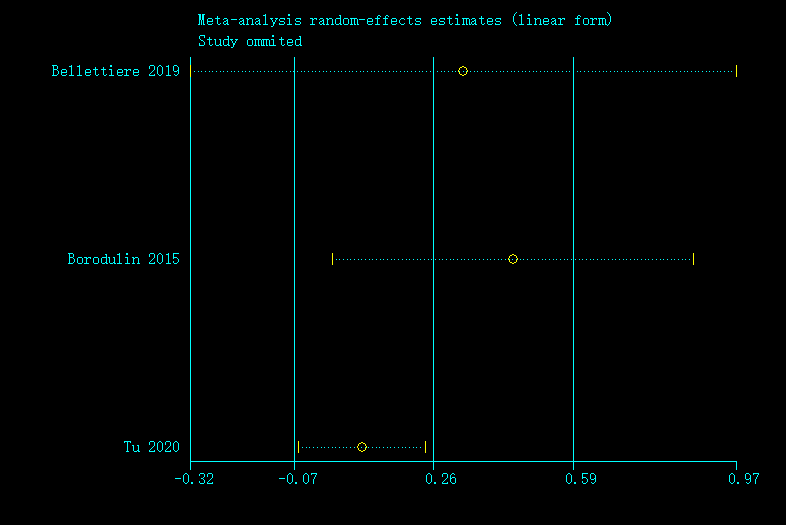


Figure S6. Sensitivity Analysis of the Association Between Highest versus Lowest Total Sedentary Time And CVD Morbidity and Mortality


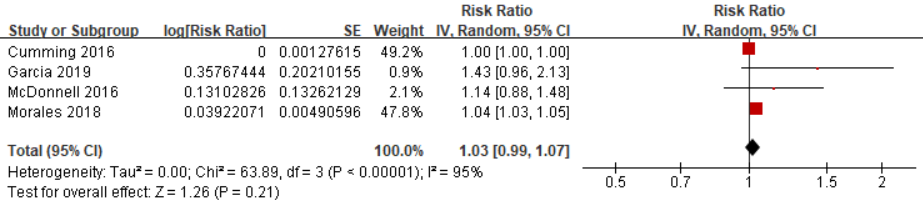


Figure S7. Forest Plot of the Association Between Highest versus Lowest Screen Time And CVD Morbidity


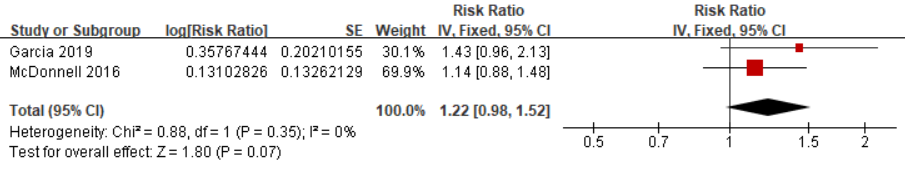


Figure S8. Forest Plot of the Association Between Highest versus Lowest Screen Time And CVD Morbidity (after excluding Cumming, 2016 and Morales, 2018)


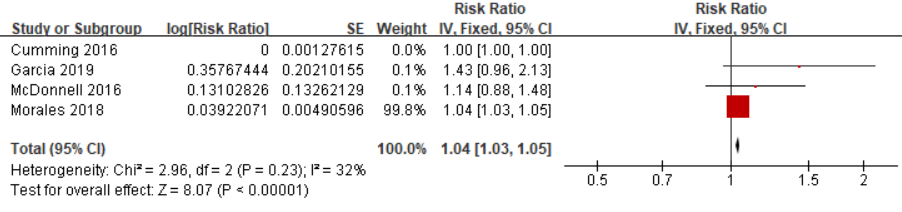


Figure S9. Forest Plot of the Association Between Highest versus Lowest Screen Time And CVD Mortality (after excluding study did not include any physical activity intensity as a covariate in the most adjusted model)


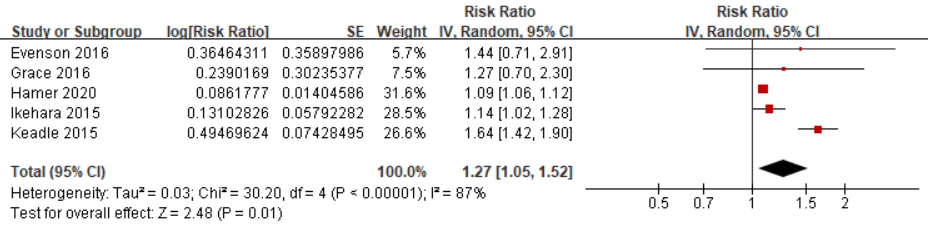


Figure S10. Forest Plot of the Association Between Highest versus Lowest Screen Time And CVD Mortality


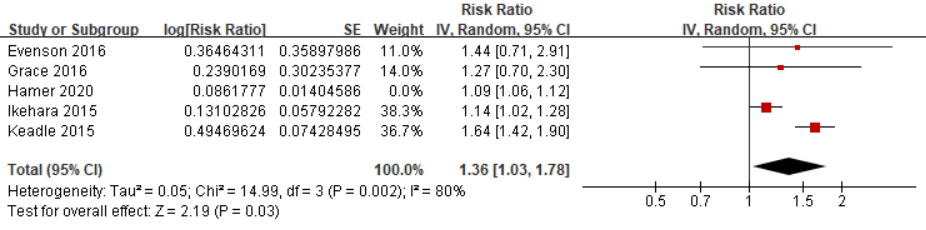


Figure S11. Forest Plot of the Association Between Highest versus Lowest Screen Time And CVD Mortality (after excluding study did not include any physical activity intensity as a covariate in the most adjusted model)


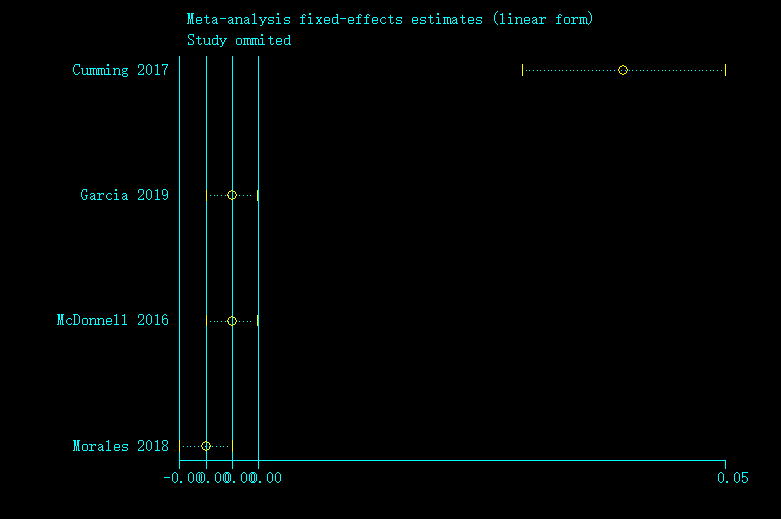


Figure S12. Sensitivity Analysis of the Association Between Highest versus Lowest Screen Time And CVD Morbidity


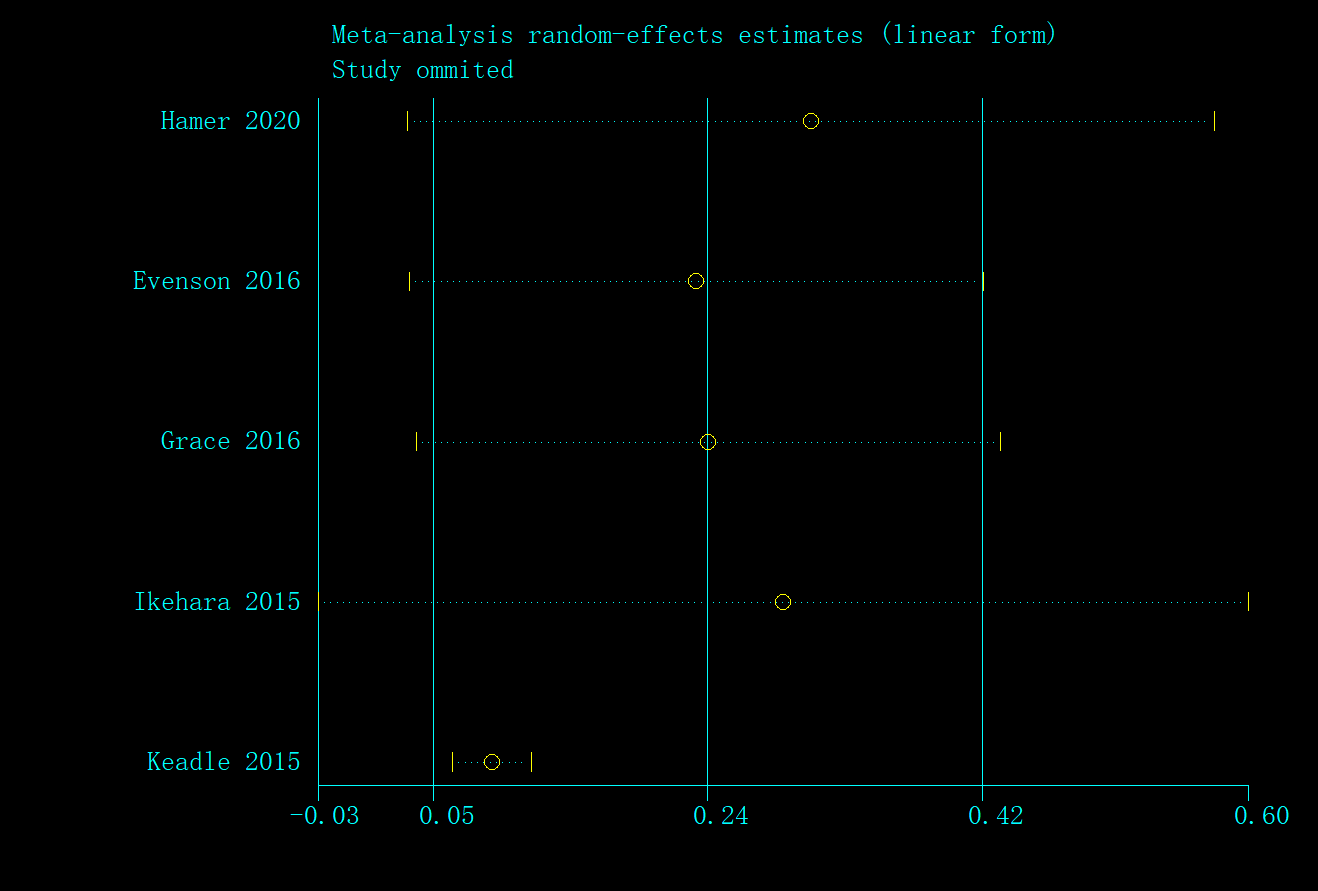


Figure S13. Sensitivity Analysis of the Association Between Highest versus Lowest Screen Time And CVD Mortality
